# Associations Between Maternal Emotional States and Plaque Accumulation in Three-Year-Old Children: A Proxy Indicator of Caregiving Quality

**DOI:** 10.7759/cureus.89444

**Published:** 2025-08-05

**Authors:** Satomi Hara, Hiroko Watanabe

**Affiliations:** 1 Department of Children and Women's Health, The University of Osaka Graduate School of Medicine, Osaka, JPN

**Keywords:** caregiving quality, maternal emotion, oral hygiene, plaque accumulation, preschool children

## Abstract

Background

Plaque accumulation can lead to dental caries and periodontal disease and may reflect overall oral health status. As mothers are often the primary caregivers for children’s oral hygiene, plaque in children may also serve as a proxy for caregiving quality. This study examined whether maternal emotional states are associated with the quality of caregiving, using children’s plaque accumulation as a proxy indicator.

Methods

We conducted a cross-sectional questionnaire survey of 150 mothers of three-year-old children who had received dental checkups within the past year. The presence or absence of plaque was assessed by a dentist. Maternal emotions-burden, anxiety, and positive emotion-were measured using the Parenting Affect Scale. Multiple logistic regression analysis examined associations between maternal emotions and plaque presence, adjusting for parental oral health behaviors.

Results

The results showed that low maternal positive emotion (adjusted odds ratio (OR): 0.76, 95% confidence interval (CI): 0.60-0.96) and low burden (adjusted OR: 0.91, 95% CI: 0.84-0.99) were associated with increased plaque accumulation.

Conclusion

These findings suggest that both low positive emotion and low perceived burden may indicate reduced emotional engagement in caregiving, potentially affecting care quality. Enhancing mothers’ emotional investment in parenting may improve consistency in daily caregiving practices related to child health.

## Introduction

Early childhood caries are one of the most common chronic diseases of children, with an estimated global prevalence of 48% [1]. In Japan, early childhood caries are diagnosed in children aged 18-36 months during dental health checkups, with prevalence rates of 1.1% and 11.8% at 18 and 36 months, respectively [2]. Although the prevalence of early childhood caries in Japan is low compared to other countries, it is increasing sharply among children aged 18-36 months [1,3]. Moreover, the prevalence rate of caries is 30.3% among preschoolers aged five years [4].

Oral health status among preschoolers is assessed during dental checkups and is based on the presence of dental caries and plaques. Dental plaque, if not regularly removed, can lead to periodontal disease and dental caries [5]. Parental involvement in daily tooth brushing is, therefore, essential [6], especially during early childhood when children cannot maintain oral hygiene independently. Furthermore, daily tooth brushing by the parents is recommended to ensure that their child’s teeth are thoroughly cleaned [3]. The rate of parental tooth brushing is 73.1% for children aged 18 months [7]. Yamada et al. [8] found that over 90% of Japanese preschoolers have their teeth brushed by their mothers, who demonstrate higher awareness of their children’s oral health than their fathers. Thus, in Japan, caries-preventive behavior for children, such as tooth brushing, is currently highly influenced by maternal actions.

The relationship of childhood caries with maternal depression and parenting stress has previously been clarified [9-11]. Moreover, preschoolers who have suffered from abuse have more tooth decay than the general population [12].

Although the association between parental behaviors and child oral health has been well documented, the influence of maternal emotional states-particularly ambivalent feelings about parenting-on caregiving quality remains underexplored. Maternal emotions are complex and can simultaneously include positive feelings, anxiety, and a sense of burden [13], which may influence caregiving engagement and consistency. However, to our knowledge, no study has examined how these emotional states relate to the quality of caregiving, as reflected by children’s oral hygiene outcomes.

This study aimed to investigate whether maternal emotional state is associated with caregiving quality, as reflected by children’s oral hygiene. Specifically, we used plaque accumulation as a proxy indicator of caregiving quality to examine whether maternal emotions relate to oral hygiene outcomes in three-year-old children.

## Materials and methods

Study design and population

This cross-sectional study included mothers of children aged three years who had received dental checkups within one year of the survey. The survey was conducted from November 2021 to September 2022. In Japan, municipalities provide dental checkups for children up to the age of 36 months. Only mothers who resided with their children were included in this study. Mothers who had difficulty reading and writing Japanese were excluded; in addition, mothers whose children had congenital, physical, or mental disorders or any disease that might influence the child’s oral health were excluded.

Survey instruments and data collection

An online survey was conducted using SurveyMonkey (Momentive Global Inc., San Mateo, CA, US) to collect data. The link to this online survey was provided to mothers in three ways: leaflets were distributed to mothers in daycares or preschools in Osaka, the questionnaire link was shared on social networks, and the questionnaire link was posted on obstetrics, gynecology, and pediatric clinic blogs in Saitama. Eligible mothers received an explanation about the study’s purpose on the website along with the questionnaire. Informed consent was obtained online through the website.

Measures

Demographics

Questions about the mothers included age, education level, marital status, employment status, financial status, and history of mental illness. Questions about the children included their age (months), gender, and whether they had siblings.

Oral Health Status (Plaque Accumulation)

In this study, a child’s oral health status was defined according to the level of plaque accumulation as determined by a dentist during a routine dental checkup at the age of three years. The mother entered the child’s dental checkup findings from the Maternal and Child Health Handbook into the web-based questionnaire.

In Japan, dental health checkups for three-year-old children are conducted as part of public health services by licensed dentists. During these checkups, the level of plaque accumulation is assessed visually on the labial surfaces of all teeth and classified into three categories: “clean” (almost no visible plaque), “little” (a moderate amount of plaque that is not clearly categorized as either “clean” or “much”), and “much” (visible plaque on nearly all teeth) [14]. The assessments are typically performed under standard lighting conditions in a clinical setting. Parents are instructed to refrain from giving their children food or drinks before the examination to ensure accurate assessment.

In this study, children with “little” and “much” plaque accumulation were included in the “presence of plaque” group, and children whose plaque accumulation was assessed as “clean” were included in the “absence of plaque” group. Although dental caries onset is a critical outcome, we selected plaque accumulation as the primary outcome because it reflects early-stage oral hygiene conditions before the development of caries. Additionally, in our study population, only 4% of children had dental caries, making it difficult to use the presence or number of carious teeth as a reliable outcome measure.

While parental oral health behaviors are essential components of caregiving, they are diverse and difficult to comprehensively measure. Therefore, plaque accumulation-being a visible and objective result of day-to-day care-was used as a proxy indicator of the overall quality of caregiving in this study.

Parental Oral Health Behaviors Toward Children

We defined parental oral health behaviors toward children as a series of actions that contributed to children’s oral health status. In this study, mothers who answered “yes” to all three items, “daily finishing toothbrushing,” “the use of fluoride varnish at home,” and “setting rules for snack time,” were included in the “sufficient oral health behaviors” group, and those who answered “no” to any of the three items were included in the “insufficient oral health behaviors” group.

Parenting Affect Scale

In this study, maternal emotions were categorized as burden, anxiety, and positive feelings, referring to the definition by Aramaki and Muto [13]. Mothers experience both positive and negative emotions, including burden and anxiety, related to parenting. The Parenting Affect Scale, which was developed by Aramaki and Muto, is a scale used to measure three emotions a mother has: positive emotion, burden, and anxiety [13]. The mothers were asked to answer a total of 21 questions using a four-point scale (1: not at all; 2: rarely; 3: sometimes; 4: frequently). Scores for “positive emotion,” “burden,” and “anxiety” range from 4 to 16, 9 to 36, and 8 to 32, respectively. Higher scores indicated high intensity of positive emotion, burden, or anxiety about parenting.

The scale has demonstrated acceptable reliability and validity in prior research; the reliability (Cronbach’s alpha) coefficient in this study was between 0.81 and 0.89 [13]. The original instrument was developed and published in Japanese [13]. We used it with permission from the original authors. A translated English version of the questionnaire is included in the appendix.

Sample size

An effect size of 0.43 was calculated with reference to the results of a previous study that showed a difference in parenting stress index scores between the having caries group and the not having caries group [15]. The preferred sample size of 170 mothers was determined using G*Power (Heinrich-Heine-Universität Düsseldorf, Düsseldorf, Germany), with an alpha value of 0.05 and power of 80% for the two-tailed test.

Data analysis

Data were analyzed using IBM SPSS Statistics Version 28.0 for Windows (IBM Corp., Armonk, NY, US), and the level of significance was set to 5%. The chi-square test, Fisher’s exact test, or Mann-Whitney U test was performed to compare participants’ characteristics and parenting oral health behaviors toward children between the two groups: “presence of plaque” group and “absence of plaque” group. We conducted a t-test or Mann-Whitney U test to compare each subscale score of the Parenting Affect Scale between the two groups. Finally, we conducted a multiple logistic regression analysis using the forced entry method to identify maternal parenting emotion factors affecting plaque accumulation in the offspring.

Variables that showed significant associations were included in multiple logistic regression models. The absence (0) or presence (1) of plaque was included as a dependent variable. Maternal parenting emotion factors served as independent variables in the model. Additionally, parental oral health behaviors toward children, variables that showed significant associations, and clinically important variables were included in the model.

Ethical considerations

The study was approved by The University of Osaka Clinical Research Review Board (Approval No. 21309).

## Results

The data of 150 mothers were analyzed in this study, with a valid response rate of 48.2%. Based on the findings of dental checkups for children aged three years in the Maternal and Child Health Handbook, the children were divided as follows: 94 (62.7%) in the absence of plaque group and 56 (37.3%) in the presence of plaque group.

Table 1 presents the characteristics of mothers and children based on the presence or absence of dental plaque. Maternal age showed a statistically significant association with dental plaque (Table 1). The prevalence of dental caries among children aged three years was 4%; 41.3% were included in the “sufficient oral health behaviors group” (Table 2). Table 3 presents the association between scores of the Parenting Affect Scale and dental plaque accumulation. Significant differences were observed between the two groups for positive emotion (p = 0.036), but no significant difference was observed between the two groups in terms of negative emotions (burden and anxiety).

**Table 1:** Comparison of mothers’ and children's demographic characteristics in the two groups N (%) or median (IQR) ^a^Mann-Whitney U test ^b^Chi-squared test ^c^Fisher’s exact test

		Plaque absence n = 94 (62.7)	Plaque presence n = 56 (37.3)	p-value
Mothers				
Age (years)		36.0 (33.0-39.0)	37.5 (34.25-42.0)	0.043^a^
Marital status	Married	93 (98.9)	57 (100)	0.627^c^
	Unmarried	1 (1.1)	0	
Educational level	High school or college	45 (47.9)	28 (50.0)	0.801^b^
	University or more	49 (52.1)	28 (50.0)	
Employment status	Employed	52 (55.3)	32 (57.1)	0.828^b^
	Unemployed or leave	42 (44.7)	24 (42.9)	
Economic status	Stable	90 (95.7)	54 (96.4)	0.600^c^
	Unstable	4 (4.3)	2 (3.6)	
Mental illness history	No	81 (86.2)	46 (82.1)	0.508^b^
	Yes	13 (13.8)	10 (17.9)	
Children				
Age (months)		51.0 (47.0-55.0)	49.5 (45.0-54.0)	0.395^a^
Gender	Boy	50 (53.2)	31 (55.4)	0.797^b^
	Girl	44 (46.8)	25 (44.6)	
Siblings	Presence	69 (73.4)	37 (66.1)	0.340^b^
	Absence	25 (26.6)	19 (33.9)	

**Table 2:** Comparison of children’s dental caries and oral health behaviors toward children in the two groups n (%) ^a^Chi-squared test ^b^Fisher’s exact test Parental oral health behaviors toward children: mothers who answered "yes" to all items marked * were included in the "sufficient oral health behaviors" group, while those who answered "no" to one or more of the items were included in the "insufficient oral health behaviors" group.

		Plaque absence n = 94 (62.7)	Plaque presence n = 56 (37.3)	p-value
Dental caries	Presence	2 (2.1)	4 (7.1)	0.140^b^
	Absence	92 (97.9)	52 (92.9)	
Oral health behavior				
Daily finishing toothbrushing*	Yes	83 (88.3)	49 (87.5)	0.884^a^
	No	11 (11.7)	7 (12.5)	
Person who primarily brushes their child's teeth (n = 149)	Mother	85 (91.4)	47 (83.9)	0.165^a^
	Father	8 (8.6)	9 (16.1)	
Have a family dental doctor	Yes	69 (73.4)	44 (78.6)	0.478^a^
	No	25 (26.6)	12 (21.4)	
Use of fluoride varnish at home*	Yes	75 (79.8)	44 (78.6)	0.859^a^
	No	19 (20.2)	12 (21.4)	
Dental checkup	Regular	67 (71.3)	42 (75.0)	0.621^a^
	Irregular or not	27 (28.7)	14 (25.0)	
Setting rules for snack time*	Yes	58 (61.7)	28 (50.0)	0.161^a^
	No	36 (38.3)	28 (50.0)	
Parental oral health behaviors toward children	Sufficient	43 (45.7)	19 (33.9)	0.155^a^
	Insufficient	51 (54.3)	37 (66.1)	

**Table 3:** Comparison of maternal parenting emotions in the two groups Mean ± SD or median (IQR) ^a^t-test ^b^Mann-Whitney U test

	Plaque absence n = 94 (62.7)	Plaque presence n = 56 (37.3)	p-value
Positive emotion	16.0 (14.0-16.0)	15.0 (13.0-16.0)	0.036^b^
Negative emotion			
Burden	20.5 ± 5.9	19.5 ± 4.9	0.301^a^
Anxiety	17.6 ± 5.0	17.4 ± 4.4	0.832^a^

Multivariable binary logistic regression analysis was conducted to determine whether maternal parenting emotions could predict plaque accumulation in children aged three years (Table 4). We found a moderate and positive correlation (r = 0.585) between the mean scores of the Parenting Affect Subscales of burden and anxiety; therefore, we included positive emotion and burden in Model 1, and positive emotion and anxiety in Model 2, as independent variables. Consequently, maternal low positive emotion and low burden were identified as the factors predicting plaque accumulation in children aged three years. Maternal anxiety was not identified as a predicting factor in Model 2.

**Table 4:** Factors associated with dental plaque accumulation in 3-year-old children in a multiple logistic regression analysis AOR: adjusted odds ratio; 95% CI: confidence interval Dependent variables: plaque absence (0) n = 94, plaque presence (1) n = 56; independent variables: maternal parenting emotion; adjusted variables: parenting oral health behaviors toward children, children's dental caries, siblings, and mother's age Model 1: positive emotion and burden were included as independent variables. Model 2: positive emotion and anxiety were included as independent variables

	Model 1			Model 2		
	AOR	95％ CI	p-value	AOR	95％ CI	p-value
Positive emotion	0.76	0.60-0.96	0.023	0.84	0.68-1.03	0.098
Negative emotion						
Burden	0.91	0.84-0.99	0.027			
Anxiety				0.95	0.87-1.03	0.948

## Discussion

We examined whether maternal emotions were associated with caregiving quality using plaque accumulation in children as a proxy indicator. Consequently, we found that low maternal positive emotions and low sense of burden were significant predictors of plaque accumulation in children.

Oral health status in children aged three years

According to a 2019-2020 survey, 48.8% of children had a family dentist, and 73.1% of parents brushed their children’s teeth daily at home. Both these values were higher (75.3% and 88.0%, respectively) for the participants in this study [7]. In the same survey, the percentage of children without dental caries in the general population was 85.6%, whereas the percentage was higher in this study at 97.6%. These findings suggest that the study sample represents a group of parents, particularly mothers, who actively engage in caregiving related to children’s oral hygiene. However, even within this relatively high-performing group, variations in plaque accumulation were observed, reinforcing its utility as a proxy indicator of day-to-day caregiving quality.

Parental oral health behaviors and plaque accumulation in children

Although inadequate parental oral health behaviors have been linked to an increased risk of early childhood caries [16], our results showed no significant differences in self-reported behaviors between the plaque and no-plaque groups. The oral health behaviors practiced by the children at daycare facilities, such as fluoride rinses or toothbrushing after meals, may have affected their oral health status. Fluoride rinses have been confirmed to be effective in preventing dental caries [3], and the national average implementation rate at facilities is 18.4% [17].

In this study, oral health behaviors were included as covariates in the analysis and not treated as primary outcomes. Owing to their limited scope and potential reporting bias, we used plaque accumulation-a more objective and cumulative outcome-as a proxy indicator of caregiving effectiveness.

Maternal positive emotion and caregiving quality

Children of mothers with low positive emotion scores were more likely to exhibit plaque accumulation. Positive parenting emotions, such as enjoyment and satisfaction, are linked to self-efficacy and mindful parenting practices [18]. Prior studies suggest that mindful parenting promotes emotional regulation, consistent caregiving, and non-judgmental awareness [19]. These factors may enhance daily health practices, including toothbrushing routines. Thus, mothers with stronger positive emotional engagement may be more likely to invest effort in their children’s daily oral care, resulting in better oral hygiene outcomes.

Maternal burden and caregiving engagement

Unexpectedly, low levels of perceived maternal burden were also associated with increased plaque accumulation. This contrasts with previous findings linking high parenting stress to poorer oral health in children [20]. One interpretation is that a low sense of burden may not always reflect ease in parenting, but rather a low level of emotional investment or caregiving engagement would. In particular, mothers reporting both low positive emotion and low burden may be less involved in their children’s daily care. In such cases, emotional disengagement may lead to reduced consistency in health-related caregiving, including oral hygiene practices. Furthermore, the persistent demands of caregiving, including daily toothbrushing, may contribute to maternal burden, particularly in societies where caregiving responsibilities are disproportionately shouldered by mothers [8]. These findings highlight the importance of promoting shared caregiving roles within families.

Maternal anxiety and oral health status in children aged three years

In this study, no significant association was found between maternal anxiety and plaque accumulation. Although previous research has demonstrated the impact of maternal anxiety on children's general health and well-being [21,22], this study focused specifically on anxiety related to parenting and child development. Parenting-specific anxiety may differ in its expression and impact from clinical anxiety symptoms. The lack of a clear association suggests that the influence of maternal anxiety on caregiving may be more complex and context-dependent. Further research should distinguish between generalized anxiety and parenting-related anxiety and examine their respective effects on caregiving behavior and child outcomes.

Limitations

This study has the following limitations. Firstly, given the cross-sectional design of the study, it does not establish cause-effect relationships between children's oral health and maternal emotions. Secondly, our study primarily focused on mothers and children attending private preschools in Osaka Prefecture. Many participants reported high levels of oral health engagement, and their children had better oral health than the national average. Third, while plaque accumulation data were obtained from official dental checkup records documented in the Maternal and Child Health Handbook, parental oral health behaviors were assessed at the time of the survey. These responses may not accurately reflect the behaviors practiced around the time of the dental checkup-approximately one year earlier-potentially weakening the association between plaque accumulation and current parenting practices. Fourth, self-reported data may be affected by social desirability bias. Fifth, although plaque was used as a proxy indicator of caregiving quality, it may not fully capture the multifaceted and dynamic nature of parenting behavior. Finally, this study focused primarily on mothers and did not account for the potential influence of other caregivers such as fathers or grandparents.

## Conclusions

The results of this study suggest that maternal emotional states, particularly positive emotion and perceived burden, influence the quality of caregiving practices as reflected by children's oral hygiene outcomes. Supporting mothers in cultivating emotional engagement in parenting-through promoting self-confidence, mindful parenting, and shared caregiving-may enhance consistency in daily health-related care. Involving fathers and other caregivers in oral health routines may also alleviate maternal burden and contribute to improved outcomes for both children and mothers. Strengthening community-based child-rearing support and interprofessional coordination, especially by public health nurses, will be critical for achieving these goals. Future research should involve more diverse populations and use synchronized assessments of children’s oral health and maternal emotional states.

## Appendices

Parenting Affect Scale

Respondents were asked to rate each item using a 4-point Likert scale:

1 = Not at all

2 = Rarely

3 = Sometimes

4 = Frequently

Items:

1. I feel that I am disconnected from society because every day is just a repetition of childcare.

2. I feel like I am raising my child all alone.

3. I get irritated because I cannot do what I want due to taking care of my child.

4. I feel like I am always making sacrifices for raising my child.

5. I get annoyed because my child makes a mess or gets things dirty.

6. Sometimes, I don’t feel affectionate toward my own child.

7. I get irritated when my child doesn’t do what I tell them to do.

8. I feel annoyed by my child.

9. I find it bothersome to think about my child.

10. I don’t know what to do about parenting.

11. I feel anxious about whether I can raise my child well.

12. I feel uncertain whether my way of raising my child is appropriate.

13. I feel like I’m not handling my child well.

14. I worry that my child may not be able to keep up with other children after entering preschool.

15. I feel like there are many things other children can do that my child cannot.

16. Compared to children of the same age, I feel that my child is immature.

17. I worry that my child may be developmentally delayed compared to others.

18. I feel that raising my child is enjoyable.

19. I think raising a child is a meaningful and wonderful experience.

20. I look forward to my child’s growth.

21. I feel that I am also growing as a person through raising my child.
